# Aptamers as quality control tool for production, storage and biosimilarity of the anti-CD20 biopharmaceutical rituximab

**DOI:** 10.1038/s41598-018-37624-1

**Published:** 2019-02-01

**Authors:** Sabrina Wildner, Sara Huber, Christof Regl, Christian G. Huber, Urs Lohrig, Gabriele Gadermaier

**Affiliations:** 1 Christian Doppler Laboratory for Innovative Tools for Biosimilar Characterization, University of Salzburg, Hellbrunner Straße 34, 5020 Salzburg, Austria; 2 Department of Biosciences, University of Salzburg, Hellbrunner Straße 34, 5020 Salzburg, Austria; 3 Technical Development Biosimilars, Global Drug Development, Novartis, Sandoz GmbH, Biochemiestrasse 10, 6250 Kundl, Austria

## Abstract

Detailed analysis of biopharmaceuticals is crucial for safety, efficacy and stability. Aptamers, which are folded, single-stranded oligonucleotides, can be used as surrogate antibodies to detect subtle conformational changes. We aimed to generate and assess DNA aptamers against the therapeutic anti-CD20 antibody rituximab. Six rituximab-specific aptamers with K_d_ = 354–887 nM were obtained using the magnetic bead-based systematic evolution of ligands by exponential enrichment (SELEX) technology. Aptamer folds were analysed by online prediction tools and circular dichroism spectroscopy suggesting quadruplex structures for two aptamers while others present B-DNA helices. Aptamer binding and robustness with respect to minor differences in buffer composition or aptamer folding were verified in the enzyme-linked apta-sorbent assay. Five aptamers showed exclusive specificity to the Fab-fragment of rituximab while one aptamer revealed a broader recognition pattern to other monoclonal antibodies. Structural differences upon incubation at 40 °C for 72 h or UV exposure of rituximab were uncovered by four aptamers. High similarity between rituximab originator and biosimilar lots was demonstrated. The most sensitive aptamer (RA2) detected signal changes for all lots of a copy product suggesting conformational differences. For the first time, a panel of rituximab-specific aptamers was generated allowing the assessment of conformational coherence during production, storage, and biosimilarity of different products.

## Introduction

Biologics or biopharmaceuticals are a new generation of medicines produced by living organisms like bacteria, yeast, or mammalian cells^1,2^. Unlike small, chemically synthesised drugs, biologics are usually large recombinant proteins which are more difficult and cost-intensive to develop and produce. Biologics are typically protected through patents; recent expirations of patent terms also allowed expansion in the field of biosimilars^3,4^. Biosimilars (or follow-on biologics in the United States) are defined as biological products highly similar to already approved biological medicines (reference medicine). In specific, those biosimilars do not show any clinically meaningful differences in terms of safety, purity, and efficacy from the reference product termed originator^5,6^.

At the amino acid sequence level, biosimilars are designed to be identical to the originator. However, proposed biosimilars and originators may still differ at the level of post-translational modifications due to differences in the highly complex production process. Such differences can potentially impact the safety, efficacy, and stability of pharmaceutical products. Therefore, detailed characterisation of the three-dimensional structure, post-translational modifications, and the aggregation behaviour of the protein is crucial to demonstrate similarity between the biosimilar and its reference product^7–9^. There are only few and rather laborious analytical methods available, like NMR or X-ray crystallography, that are able to detect subtle changes in the tertiary structure of proteins. Another method to monitor potential differences is the use of monoclonal antibodies specific to the target biologic. This can however be restricted by the availability of appropriate antibody panels and also typically involves animal experiments for initial antibody generation^10–12^.

An alternative approach to overcome these limitations is the application of aptamers. Aptamers, which are single-stranded DNA or RNA oligonucleotides with a specific three-dimensional structure, are typically obtained using the *in vitro* selection process termed systematic evolution of ligands by exponential enrichment (SELEX). Aptamers are able to bind various targets, such as proteins, small molecules, glycoproteins or even cells^13–15^. As they present a defined fold which can recognise a target with high affinity and specificity, they can be used as surrogate antibodies^16–18^. Unlike antibodies, aptamers can also be generated for targets that do not elicit immune responses as well as for toxic targets. A study from Zichel *et al*. demonstrated that aptamers were able to detect differences between native and heat-treated thrombin, whereas antibodies failed to detect them^17^. The authors therefore suggested that aptamers have the potential to improve quality control during production and storage of proteins. Aptamers were also proposed to be a potent method for comparison of originator and biosimilar. However, so far and to the best of our knowledge, no study covering this application has been published.

Rituximab, one of the most important biologics in cancer treatment, is a chimeric monoclonal anti-CD20 antibody produced in Chinese hamster ovary (CHO) cells^18,19^. It was the first monoclonal antibody (mAb) for the treatment of lymphoma approved by the Food and Drug Administration (FDA) in November 1997 and by the European Medicines Agency (EMA) in June 1998^19,20^. The trade names are MabThera (Roche, Basel, Switzerland) for the European market and Rituxan (Biogen Idec Technologies, Cambridge, US) for the US market^21^. With 8.6 billion US$ global sales in 2013, rituximab represents one of the best-selling biologics worldwide^22^. The antibody binds specifically to human CD20, an antigen expressed on the surface of B-lymphoid cells and is approved for the treatment of non-Hodgkin lymphoma (NHL)^23^. NHL is the tenth most common cancer worldwide and the incidence rate is highest in Northern America^24,25^.

Due to the high worldwide prevalence of NHL, the need for efficient therapeutic options is constantly growing. Several studies have shown that rituximab in combination with chemotherapy regimens leads to lymphoma regression and significantly improves treatment outcome^26–28^. Nevertheless, access to rituximab has been limited, especially in countries with restricted financial resources. The most common access barriers are a consequence of limited insurance coverage, treatment guidelines, or patient comorbidities. These facts led to the development of more affordable biosimilars^29^. For approval of biosimilars, similarity between the biosimilar and the reference molecule in terms of safety, efficacy and quality needs to be demonstrated.

The aim of this study was to select and verify DNA aptamers against rituximab using the *in vitro* selection process SELEX. Six DNA aptamers reactive with rituximab were identified using ELASA. Binding affinities in the nanomolar range were determined and structural analyses revealed B-DNA helices and quadruplex structures. Robustness of the test assays was verified and specific binding mainly to the Fab fragment of rituximab was revealed. Selected aptamers were able to detect structural changes of thermally or UV light stressed rituximab. Analysis of different rituximab biosimilar candidates revealed a high similarity between the products, while one aptamer was able to reveal a structural difference between the originator and a proposed copy product.

## Results

### *In vitro* selection of rituximab specific DNA aptamers

**Figure 1:**
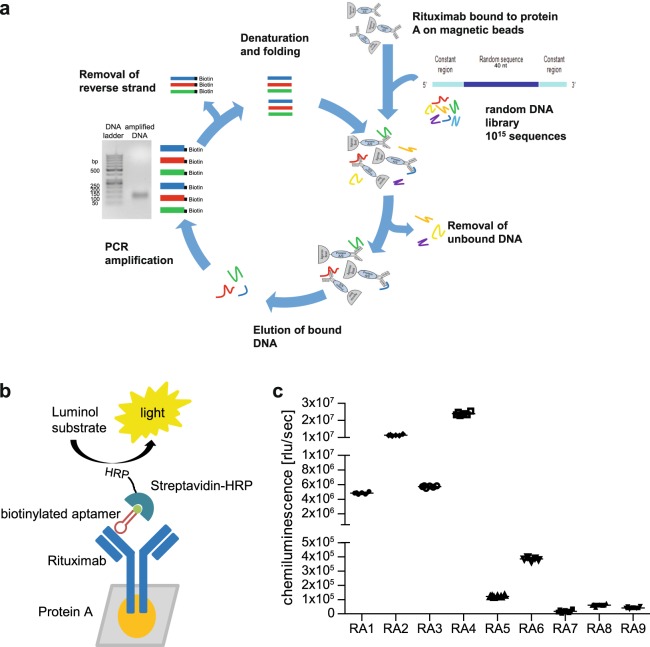
*In vitro* selection and binding of aptamers. (**a**) Schematic representation of the SELEX process used within this study. (**b**) Schematic illustration of the enzyme linked apta-sorbent assay (ELASA) setup. (**c**) Folded biotinylated aptamers (RA1-RA9, c = 500 nM) were tested for rituximab binding. Bound aptamers were detected by streptavidin-HRP, chemiluminescence ELISA substrate was used for detection and luminescence was measured. Measurements were performed in six replicates; means and standard errors of the mean are given.

A DNA-library consisting of 10^15^ different single-stranded oligonucleotides with a random part of 40 nucleotides in length was used for selection of aptamers against the therapeutic IgG1 antibody rituximab. *In vitro* selection was performed by eight recurring incubations of rituximab-coated protein A magnetic beads using the folded single stranded oligonucleotides (Fig. 1a). Stringency of the SELEX process was increased in the last selection rounds by decreasing the amount of DNA incubated with the beads, increasing the number of washing steps and decreasing the number of PCR cycles. Additionally, a negative selection round was carried out with uncoated protein A magnetic beads before the last cycle. After cloning the DNA fragments of SELEX cycle eight into a cloning vector, plasmids from 50 clones were obtained and sequenced. Analysis of the 40 nucleotide random part revealed fifteen different rituximab aptamer (RA) sequences with occurrences ranging from 1–12 times (Supplementary Table S1).

### Six aptamers efficiently bound to rituximab

For the first binding experiments, sequences occurring ≥2 (RA1–9) were obtained as 5′-biotinylated aptamers and assessed using an enzyme-linked apta-sorbent assay (ELASA). For this, biotinylated aptamers were incubated with protein A immobilised rituximab and the interaction was detected using streptavidin-HRP (Fig. 1b). Highest signals were observed with RA2 and RA4, binding capability was medium for RA1 and RA3, while RA5 and RA6 showed lower binding (Fig. 1c). RA7–9 demonstrated very low signals and those sequences were therefore not further pursued in this study. Titrations of RA1–6 ranging from 1.95–1000 nM were performed to identify optimal working concentrations. All aptamers bound to rituximab in a dose-dependent manner, though with different kinetics (Supplementary Fig. S1). Based on these results, aptamer concentrations of 25–50 nM were found to efficiently detect rituximab in the ELASA and were consequently employed in subsequent experiments. Aptamer affinity was measured by surface acoustic wave and K_d_ values ranging from 354–887 nM were experimentally determined (Table 1).

**Table 1:** Aptamer sequences reactive to rituximab.

Name	Aptamer sequence (5′-3′)	Occurrence	G-score*	GC-content (%)	K_D_ [nM]
RA1	C GG C GG G GG GA GG ATTGTGGTCTGCTCATGGCTGCCGTTT	4	20	65.0	683
RA2	T GG GGGTA GG ATTGT GG TTGGCTTTAATTGCTTTGGTGGT	4	18	47.5	394
RA3	GGG GG TGA GG ATTGT GG TTT GG CTTATTGGTTTGCTGGTG	12	19	52.5	713
RA4	TATACTGGGCCGTGCGTGACTTTTCCGTGCTGCATGAGAG	3	—	55.0	354
RA5	GGCCGGTAGATGG GG AATC GG TTTC GG TGG GG CTAGGGAC	2	20	65.0	n.d.
RA6	CGT GG GTGG GG ATTGT GG TTT GG CTGATGGGGTGCTGGTT	2	19	60.0	887

Guanine bases (G) predicted to be involved in the formation of G-quadruplex structures are shown in bold and underlined.

*G-score indicates the likelihood of a G-rich sequence to form quadruplexes. Maximum G-score value with the recommended setting is 105. n.d. = not determinable.

### *In silico* prediction of aptamer folds

**Figure 2:**
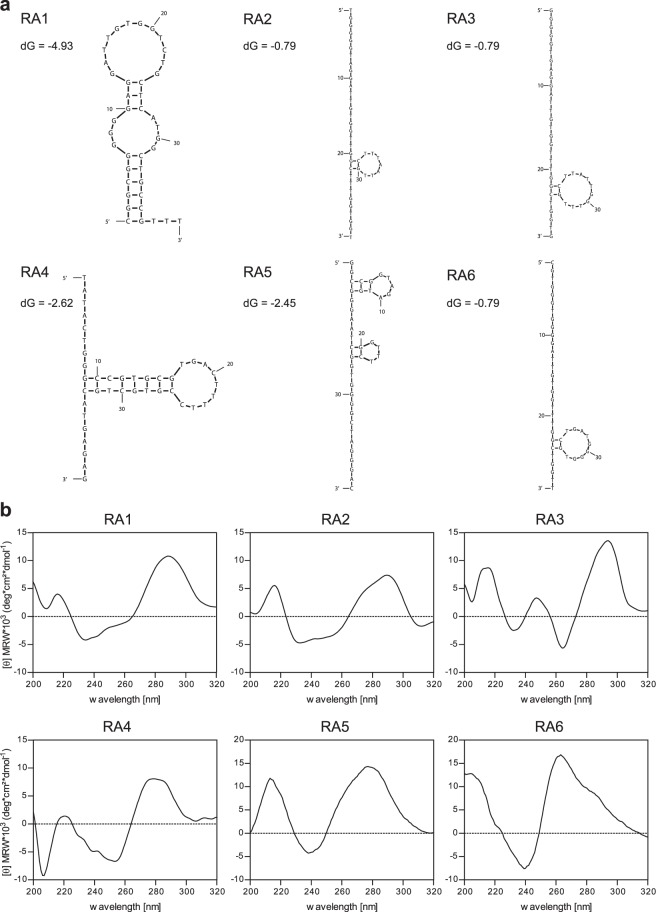
Aptamer secondary structure prediction and determination. (**a**) Secondary structures and negative energies of folding (dG) were obtained using the Mfold online tool. (**b**) Circular dichroism spectroscopy of non-biotinylated aptamers folded in binding buffer at 20 °C.

It is well established that aptamer stability depends on nucleotide composition and resulting three-dimensional structure. In specific, an elevated GC-content was noted for RA1, RA5, and RA6 (Table 1). *In silico* analysis of the sequences was performed using the online tool Mfold and most probable structures with the lowest free energy (dG) were selected and are displayed in Fig. 2a. These predictions suggested hairpin structures of varying length and loop formations with lowest energies of folding noted for RA1 (dG = −4.93 J) and RA4 (dG = −2.62 J). In addition, the capacity to form G-quadruplex structures was studied using the web-based QGRS Mapper program. Interestingly, results indicated that all aptamers except RA4 could adopt G-quadruplex structures and suggested guanine bases involved in G-tetrads are highlighted in Table 1. Based on the calculated G-score values which indicate the probability of a G-rich sequence to form a quadruplex structure, rituximab aptamers reached values of 18–20 out of 105 maximum score as a consequence of four times staples of two guanines.

### Circular dichroism spectroscopy indicates B-DNA and quadruplex structures of aptamers

The secondary structure was further experimentally investigated using circular dichroism spectroscopy (Fig. 2b). For this experiment, non-biotinylated aptamers were folded in binding buffer before analysis. CD spectra of aptamers RA1, RA2, RA4 and RA5 were rather similar showing a positive maximum around 280 nm and a negative minimum around 245 nm indicative of a B-DNA helix^30,31^. In contrast, the CD spectrum of RA3 revealed a positive maximum at 295 nm and a negative minimum at 260 nm, characteristic for an anti-parallel quadruplex structure. A typical parallel quadruplex structure with a positive maximum at 260 nm was observed for RA6^32,33^.

### Aptamers demonstrate robustness to minor changes in buffer composition and aptamer folding

**Figure 3:**
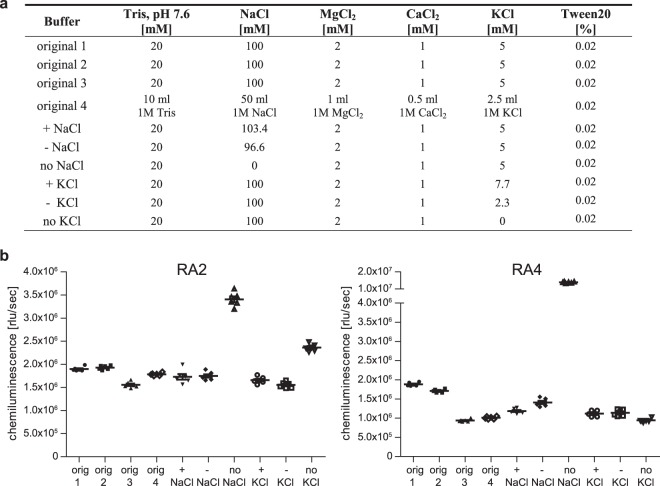
Influence of different binding buffers in ELASA. (**a**) Overview on binding buffer compositions. Original 1 and 2, original 3 (prepared by different operator), original 4 (prepared from stock solutions) and buffers with more, less or no NaCl or KCl. (**b**) Biotinylated RA2 and RA4 were folded in the different binding buffers as indicated above. Mean and standard errors of the mean are represented in the graph.

To investigate the robustness of aptamers within our ELASA setup, different aptamer buffers and folding conditions were analysed. Experiments were exemplarily carried out with RA2 and RA4, which showed highest binding affinities. To determine the influence of buffer composition and potential differences during preparation (e.g. weighing errors), a set of different buffers varying in preparation or ionic strength were assembled (Fig. 3a). Minor to moderate differences were observed when buffers were prepared in different ways or by another researcher (Fig. 3b). Minor variations in ionic strength showed no significant effect on aptamer binding. However, omitting sodium chloride triggered a considerable increase in detection signal, while potassium chloride led only to a moderate enhancement for RA2, a result of unspecific interaction of negatively charged DNA with positively charged protein surface areas.

**Figure 4:**
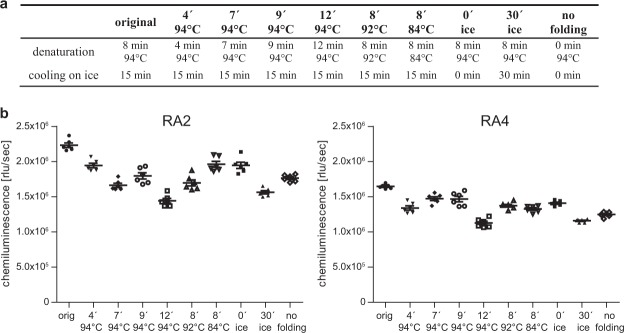
Investigation of different folding conditions in ELASA. (**a**) Folding conditions differing in denaturation or cooling used in the ELASA experiment. (**b**) Biotinylated RA2 and RA4 were folded in binding buffers under different folding conditions. Typically, aptamers were denatured at 94 °C for 8 min, followed by a cooling step on ice for 15 min and a step at ambient temperature for 10 min (orig = original protocol). Mean and standard errors of the mean are represented in the graph.

In addition, the influence of aptamer folding conditions was investigated. Aptamers were typically denatured at 94 °C for 8 min, followed by a cooling step on ice for 15 min and a step at ambient temperature for 10 min (designated as original protocol). To test the robustness of aptamer folding, denaturation temperature, time as well as the subsequent cooling steps were amended (Fig. 4a). Generally, the original protocol resulted in highest signals but only minor to moderate signal decreases were found for the other folding protocols (Fig. 4b). Highest impact was detected upon an extended denaturation step for 12 min at 94 °C. Interestingly, completely omitting the folding step did not result in failure to bind rituximab, but showed only decreased binding, similar to those aptamers folded under different conditions.

### RA1–5 present high specificity for rituximab

**Figure 5:**
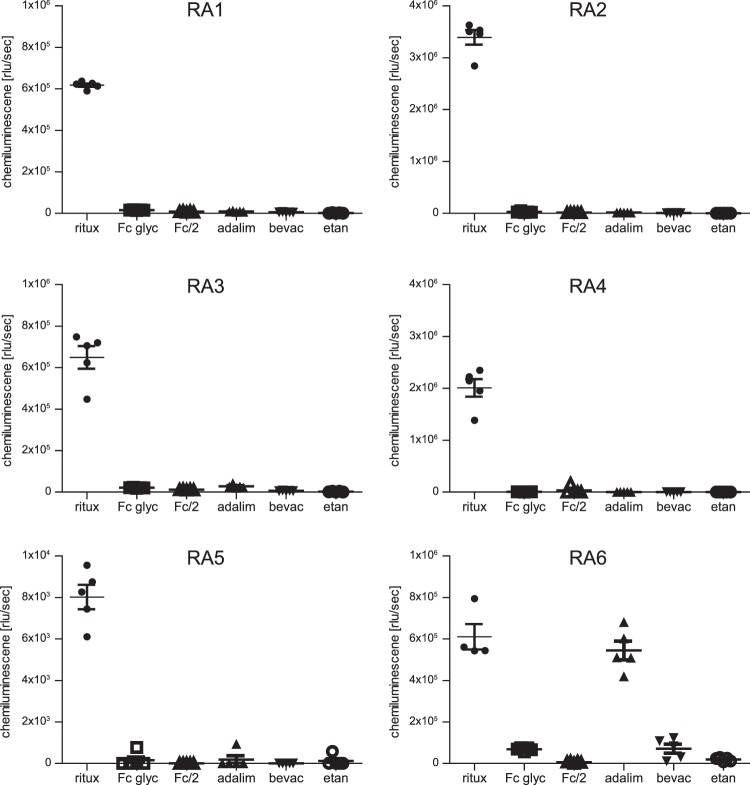
Investigation of aptamer specificity using ELASA. Rituximab (ritux), glycosylated Fc of rituximab (Fc glyc), recombinantly expressed Fc/2 (Fc/2), adalimumab (adalim), bevacizumab (bevac), and etanercept (etan) were tested using biotinylated RA1-RA6. Means and standard errors of the mean of five replicates are shown in the graph.

To investigate aptamer specificity, rituximab, Fc fragments and other biopharmaceuticals were tested in ELASA. Two different Fc fragments of rituximab were analysed, (i) glycosylated Fc obtained after FabRICATOR (IdeS) digest and (ii) recombinant non-glycosylated Fc/2 obtained from *E. coli*^34^. In addition, adalimumab (IgG1), bevacizumab (IgG1), and etanercept (Fc part of IgG1) were investigated. All aptamers except RA6 exclusively recognised full-length rituximab pointing at their high specificity. RA6 also efficiently recognised adalimumab; low but positive reactivity was also observed for bavacizumab and the glycosylated Fc fragment (Fig. 5).

### Aptamers were able to detect changes induced by thermal stress and UV exposure

**Figure 6:**
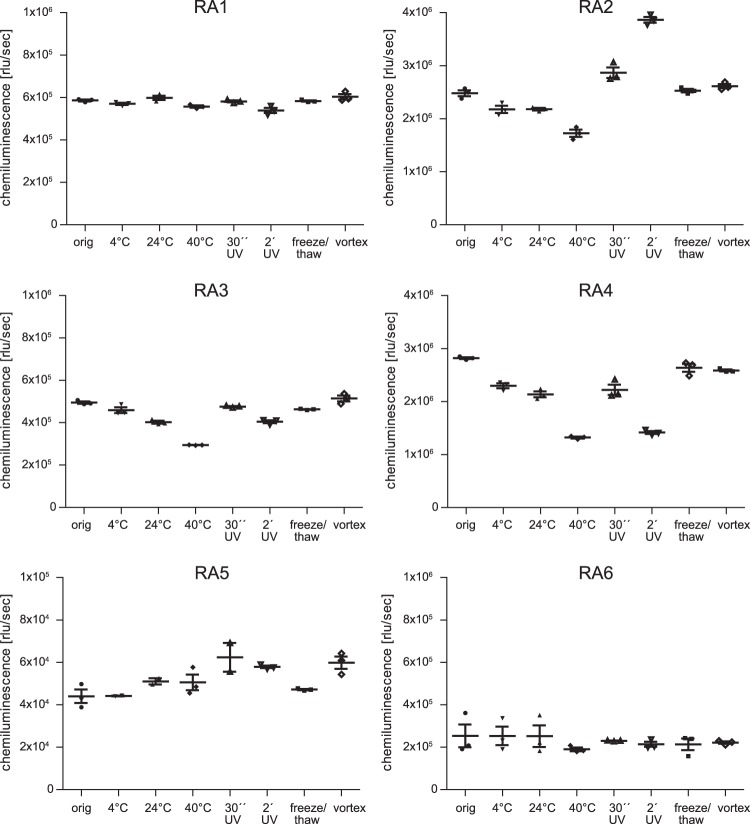
Analysis of differently stressed rituximab using ELASA. Prior to protein A binding, aliquots were exposed to different temperatures for 72 hours, UV light or mechanical stress. Mean of triplicates and standard error of mean are depicted.

In order to assess the use of aptamers as potential tools for quality control during protein production and storage, differently stressed rituximab samples were investigated by ELASA. For this purpose, aliquots of rituximab were stored at different temperatures, exposed to UV light or stressed mechanically. All aptamers except RA1 and RA6 were able to detect differences between the untreated and treated rituximab samples (Fig. 6). Storage of proteins at 40 °C for 72 hours led to reduced binding of RA2-RA4, while signals remained similar when kept at RT or 4 °C or 24 °C for the same time. Interestingly, UV exposure of rituximab resulted in increased but also decreased signals while mechanical stress (vortexing) and repeated freeze-thaw cycles did not considerably influence aptamer binding.

### Investigation of different rituximab biopharmaceuticals

**Figure 7:**
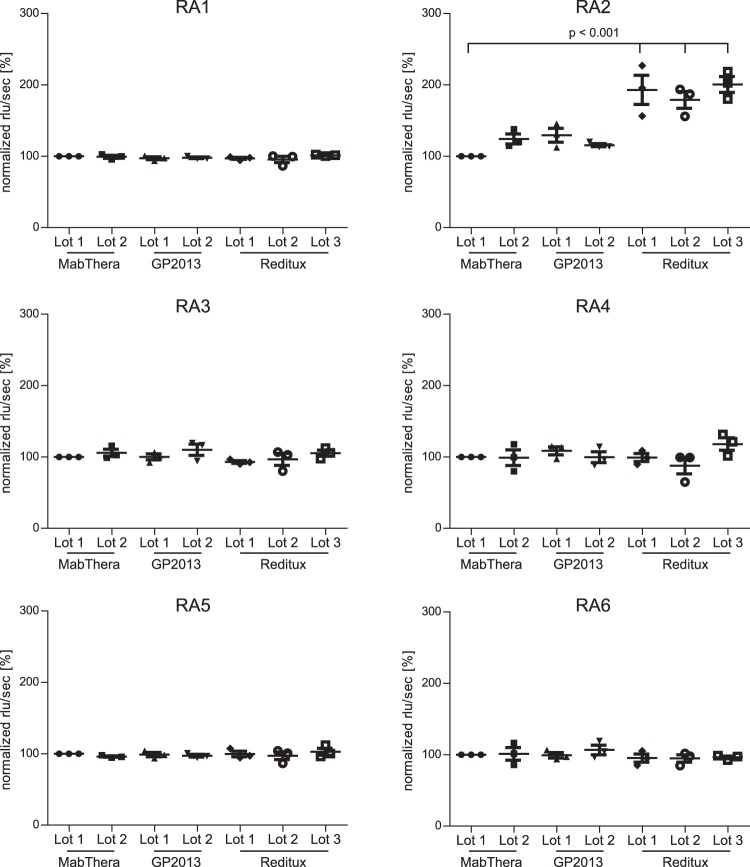
Comparison of different rituximab biopharmaceuticals. Two lots of rituximab originator (MabThera), two lots of the biosimilar (GP2013) and three lots of the copy product (Reditux) were investigated by ELASA and data were normalised to percentages based on 100% reactivity to MabThera lot 1. Data represent mean values from 3 independent ELASA experiments with 6 replicates each. Mean and standard error of mean are depicted.

Rituximab aptamers were further employed to investigate the similarity between different batches of the originator (MabThera, Roche), a proposed biosimilar (GP2013, Sandoz) as well as a copy product (Reditux, Dr. Reddys) (Fig. 7). The assay was performed in triplicates on independent days and given values represent the mean of six replicates of each measurement (Fig. 7). For statistical analysis, all rituximab samples were compared with lot 1 from MabThera using one-way ANOVA. Generally, a high degree of similarity was found for the different rituximab products. However, while RA1, RA3–6 showed no statistically significant difference, RA2 was able to reveal a difference in the signals towards the copy product Reditux. Slightly increased signal intensities were also observed for different lots of MabThera and the proposed biosimilar GP2013, which were however beyond statistical significance.

## Discussion

Biologics or biopharmaceuticals represent an important branch of the pharmaceutical industry. Patents for some well-established biopharmaceuticals have expired or are approaching expiration. Currently, there are 38 approved biosimilars in the EU and 7 in the US^35–37^. Several technologies are accepted for characterisation of the physicochemical and biological properties of biosimilars^17^. However, methods to investigate and compare the tertiary structure of proteins are rather limited and laborious. This encouraged us to employ aptamers for monitoring conformational similarities between different biologics and as a quality control tool for the detection of differently treated samples. In our study, we identified the first panel of aptamers reactive against the monoclonal anti-CD20 antibody rituximab using the *in vitro* selection process FluMag SELEX^38^. Most studies use oligonucleotide libraries with a length of 20–80 nucleotides; for our study we chose a 40 nucleotide random part to obtain stable structures with high affinity to the protein target^15,39,40^. For selection of specific aptamers, rituximab was immobilised on the surface of magnetic beads. Several immobilisation methods like capillary electrophoresis, flow cytometry, or electrophoretic mobility shift assays are available. The use of magnetic beads is however easy to handle, adaptable for many kinds of targets and requires only small amounts of target^15,41–43^. To avoid potential loss of aptamers during initial counter selection, we chose to perform this step later based on an optimized protocol for FluMag SELEX by Stoltenburg *et al*.^38^.

In an initial step, binding of nine aptamers with ≥2 occurrence was investigated by ELASA^44^. Even though all nine tested aptamers showed similar sequence occurrences of 2–12 times, RA7–9 were negative in binding properties suggesting that their high representation after cloning might be due to an amplification bias during PCR. Indeed, it was shown that some motifs (e.g. GC-rich) are preferred by DNA polymerases, especially when several templates are amplified simultaneously^45,46^. The aim of our study was the generation of relatively small aptamers consisting of 40 nucleotides. Therefore and owing to the fact that recent studies suggested limited influence on aptamer functionality upon primer binding site truncation, our initial ELASA-based studies were carried out using only the random sequence part^39,47,48^. In the literature, little influence of 5′ or 3′-labelling was noted and the impact of biotinylation was found to be lower compared to other labellings^48,49^. Using protein A for immobilisation of the Fc antibody part considerably enhanced the replicate reproducibility in the assay compared to direct coating of rituximab^49^. In summary, we were able to select six aptamers reactive to rituximab showing K_d_ levels in the upper nanomolar range.

GC-rich sequences were proposed to stabilise DNA structures and all aptamers except RA2 showed elevated contents^50^. However, sequences were especially enriched in repetitive blocks of two guanine bases, and such motifs are able to form G-quadruplex structures^50–52^. Indeed, *in silico* prediction and more importantly experimental CD data concurrently suggest an anti-parallel and a parallel quadruplex structure for RA3 and RA6, respectively^32,33^. The predicted quadruplex structures for the other aptamers (except RA4) could not be experimentally confirmed by CD which rather suggested a B-DNA form^30,31^. In general, formation of quadruplex structures strongly depends on buffer composition, in particular the concentration of cations (Na^+^, K^+^, Mg^2+^) and pH. Generally, increasing the Na^+^ and K^+^ concentration leads to a stronger CD signal of anti-parallel quadruplex structures, and Mg^2+^ cations have a positive effect on the stability of the aptamer structure^53^. For consistency, we analysed the aptamers in the same buffer as used for aptamer folding in the ELASA experiments. Besides, secondary structures were also predicted using Mfold, which suggested various stem-loop structures. Of note, negative folding energy showed very low values for RA3 and RA6, which is in line with our experimental data revealing both as G-quadruplex structures. Interestingly, also a low dG was observed for RA2 even though it shows highest binding affinity. Tools for nucleotide fold prediction typically rely on classical base pairing while alternative structures like G-quadruplexes seem currently challenging to adequately predict. To date, the most reliable method is (co)-crystallisation of aptamers but there is still limited experimental data available due to the resource intense procedure^54^. Once tools have enough reference data for accurate prediction of 3-dimensional structures analogous to protein modelling, there is enormous potential for the aptamer technology in terms of *a priori* selection and design^55^.

Biopharmaceuticals need to fulfil stringent quality standards for approval by the authorities. Methods used to assess the product quality thus need to demonstrate sufficient robustness regarding different buffer batches, days, laboratories or analysts^56^. Based on the ELASA results, we conclude that the herein presented setup with aptamers results in comparable reading outputs with respect to minor differences in buffer composition and folding conditions. It has to be noted that - analogous to ELISA experiments - most reliable results are obtained when comparing different analytes using identical buffer conditions applied within the very same assay. Especially RA2 showed high robustness if minor weighing mistakes of chemicals were considered. Completely omitting NaCl, and to a lesser extent KCl, led to a tremendous increase in signal which is however due to unspecific binding where negatively loaded phosphate groups of the DNA can bind to positively charged protein surfaces irrespective of the aptamer sequence^57,58^.

Five aptamers exclusively recognised rituximab suggesting an interaction within the antigen binding sites. Solely RA6 additionally bound to adalimumab and bevacizumab as well as to the glycosylated Fc fragment of rituximab. Constant regions of IgG antibody subclasses are highly conserved^59,60^, and indeed rituximab and investigated antibodies’ sequence identities of constant domains range from 98–100%, while variable region identities are only between 44–62%. As adalimumab was well recognised by RA6, either a conserved stretch within the CH1/CL domain or the hinge region might be a potential interaction site. Although the amino acid sequences are highly similar, the hinge region shows high structural flexibility and could therefore also lead to different epitopes^60^.

Development and production of biologics are highly complex processes, where changes in the manufacturing process can impact the structure and/or function of a protein. A panel of powerful tools is used to characterise such products, but minor conformational changes are often difficult to detect by bioanalytical methods^10^. In a pioneer study by Zichel *et al*., aptamers were able to detect differences between native and heat-treated thrombin, whereas antibodies failed to reveal them^17^. In our study, four rituximab aptamers showed differences predominately in binding to samples stored at 40 °C or exposed to UV light. As those stress conditions led to different recognition pattern, the involvement of distinct rituximab epitopes can be anticipated. From a quality control point of view, RA2, RA3 and RA4 seem adequate as an orthogonal method to detect changes during production and storage. In comparison to antibodies, large numbers of aptamers can be generated against any given target as immunogenicity is no prerequisite for development. With respect to rituximab, only a limited number of anti-idiotypic antibodies are commercially available (www.abnova.com; www.biorad.com). Advantages of aptamers are the *in vitro* selection process, chemical synthesis avoiding batch-to-batch variability of antibodies, introduction of modifications and functional groups as well as their cost-efficiency^61^. In contrast to antibodies, aptamers can easily re-adopt their fold upon denaturation without loss of activity.

Aptamers could thus also be implemented as additional analytical method for development and approval of biosimilars^17^. For approval of biosimilars, high similarity to the originator molecule in terms of safety and efficacy needs to be demonstrated^62,63^. Besides an array of analytical methods^3^, there is still a gap in rapid and routine monitoring of minor conformational changes in biologics that might not be detectable by circular dichroism or Fourier transform infrared spectroscopy. Using six aptamers in our ELASA setup, no significant differences between the originator MabThera and the proposed biosimilar candidate GP2013 were detectable supporting the high similarity of the products. Interestingly, RA2 coherently revealed increased signals for all three batches of Reditux, the “copy product” or “similar biologic” from the Indian market in three independent experiments. Mass spectrometry based analyses of these products revealed only little differences between Reditux and the originator molecule MabThera apart from lysine variants at the C-terminus (Supplementary Fig. S2). However, within the Fab fragment, only minor discrepancies beyond statistical significance were identified for Reditux mostly involving pyro-glutamate formation, a common modification observed in mAbs involving charge variants^64^. As aptamers specifically recognize the Fab fragment of rituximab, the different signals of RA2 towards Reditux indicate recognition of structural variations that could not be resolved by mass spectrometry. In this respect, it should be noted that the regulatory process for biosimilar approval in India differs from the process in the EU or US^65^. Reditux was already approved 2007 in India, comparison of efficacy and safety regarding MabThera and Reditux and phamacokinetic studies have been performed thereafter^66,67^. We now showed for the first time that aptamers are also suitable for comparison of originator and biosimilars in their native conformation. In specific, RA2 can be regarded as a highly specific and robust aptamer revealing differences upon prolonged storage or UV exposure and is able to detect conformational variations in native biopharmaceutical products. In summary, the first aptamer panel against the therapeutic antibody rituximab was generated. Based on our results, we suggest including the aptamer technology as orthogonal analytical approach in the portfolio of analytical techniques for characterisation of biologics.

## Material and Methods

### Immobilisation of rituximab to protein A magnetic beads

700 µl (1.89 × 10^9^ beads) of protein A magnetic beads (Dynabeads® Protein A, Life Technologies AS, Oslo, Norway) were washed with 500 µl 137 mM NaCl, 2.7 mM KCl, 10 mM Na_2_HPO4, and 1.8 mM KH_2_PO4, 0.05% v/v Tween 20 (PBST). 240 µg of rituximab (MabThera, Roche, Basel, Switzerland) in 700 µl PBST were added and incubated for 4 h at room temperature with gentle rotation. The supernatant was removed and beads were washed 3 x with PBST. Binding efficiency of rituximab to the magnetic beads was verified by reducing sodium dodecyl sulfate polyacrylamide gel electrophoresis.

### *In vitro* selection of DNA aptamers using the FluMag-SELEX

The random unlabelled single-stranded DNA (ssDNA) library was obtained from IBA GmbH (Göttingen, Germany). The library consisted of oligonucleotides with 40 randomised bases flanked by 18 bases required for PCR amplification (5′-ATACCAGCTTATTCAATT-N_40_-AGATAGTAAGTGCAATCT-3′). For each cycle, an aliquot of 80 µl rituximab-coated protein A magnetic beads was washed eight times with 20 mM Tris-HCl pH 7.6, 100 mM NaCl, 2 mM MgCl_2_, 5 mM KCl, 1 mM CaCl_2_, 0.02% v/v Tween 20 (binding buffer). For initial aptamer folding, 2 nmol of the ssDNA library was incubated in 500 µl binding buffer at 94 °C for 8 min, immediately cooled on ice for 15 min and then kept at room temperature for 10 min. The rituximab-coated magnetic beads were incubated with the folded ssDNA pool with gentle shaking. After 1 h at room temperature, the supernatant was removed and beads were washed 5 times with 500 µl binding buffer. Bound DNA was eluted three times with 200 µl 40 mM Tris HCl pH 8.0, 10 mM EDTA, 3.5 M urea, 0.02% v/v Tween 20 (elution buffer) at 80 °C for 8 min. Eluted ssDNA was precipitated with 1/10 volume of 3 M sodium acetate pH 5.2 and 2.5 volumes of cold 100% ethanol. One fifth of the eluted ssDNA was amplified in a large-scale PCR amplification in 10 parallel reactions of 100 µl containing 0.5 µM forward primer (5′-ATACCAGCTTATTCAATT-3′), 0.5 µM biotin-labelled reverse primer (5′-biotin-AGATTGCACTTACTATCT-3′), 0.2 mM dNTP (Promega, Madison, USA). 1 U Q5® High Fidelity Polymerase (New England Biolabs, Ipswich, USA), Q5 Reaction Buffer were added and amplification was improved by addition of the Q5 High GC Enhancer. PCR was performed at 94 °C for 5 min and 30 cycles with 30 s at 94 °C, 30 s at 50 °C, 30 s at 72 °C. Obtained dsDNA was bound to streptavidin magnetic beads (Dynabeads® M-280 Streptavidin, Life Technologies AS, Oslo, Norway) and the forward DNA strand used as template in the next cycle, was obtained by a denaturation step according to Rouah-Martin *et al*.^68^. Cycles were repeated six times, whereby stringency was enhanced by decreasing the amount of DNA for binding to rituximab to half, doubling the number of washing steps and decreasing PCR cycle numbers to 20. In cycle seven, a counter selection against protein A magnetic beads was performed to avoid unspecific binding. After this, a final cycle with the rituximab coated beads was performed.

### Cloning and sequencing

Selected oligonucleotides from cycle eight were amplified with the forward and unmodified reverse primer (5′-ATACCAGCTTATTCAATT-3′) and GoTaq® DNA Polymerase (Promega, Madison, USA). The PCR product was cloned into the pGEM®-T easy vector (Promega) and transformed into the *E. coli* NovaBlue. Fifty colonies were selected, plasmid DNA was purified using EZ-10 spin column plasmid DNA Miniprep Kit (Bio Basic Inc., NY, USA) and the aptamer sequence of each clone determined (Eurofins Genomics, Ebersberg, Germany). DNA sequences were analysed with Clustal Omega^69^. To keep aptamers short, nine selected aptamer sequences without primer binding regions were synthesised as non-labelled and 5′-biotin-lablled oligonucleotides (Eurofins Genomics).

### Enzyme linked apta sorbent assay (ELASA)

50 µl of recombinant Protein A (4 µg/ml) (Thermo Fisher Scientific, Waltham, MA, USA) diluted in 0.2 M ammonium bicarbonate buffer pH 9.4 was coated onto white Maxisorp FluoroNunc/LumiNunc 96-well ELISA plates (Thermo Fisher Scientific) for 3 h at room temperature. Plates were washed three times with 200 µl 1x PBST and blocked with 200 µl 1x PBST, 0.5% w/v BSA for 2 h at room temperature. After blocking, the plates were incubated overnight with 50 µl rituximab (MabThera lot N7075B10, Roche) (4 µg/ml in PBS) at 4 °C. The plates were washed three times with 200 µl of 137 mM NaCl, 2.7 mM KCl, 10 mM Tris, pH 7.4, 0.05% v/v Tween 20 (TBST). Biotinylated aptamers (c = 4000 nM) in binding buffer were heated to 94 °C for 8 min, cooled on ice for 15 min and kept at room temperature for 10 min. 50 µl of folded aptamers diluted to the respective concentration in binding buffer were added and incubated at room temperature protected from light for 2 h. For determination of titration curves, folded aptamers were gradually diluted (1000 nM to 1.95 nM) in binding buffer. After three washing steps with 200 µl TBST, bound aptamers were detected with 50 µl 1:2000 diluted horseradish peroxidase-conjugated streptavidin (Caltag Laboratories, Carlsbad, CA, USA). BM Chemiluminescence ELISA Substrate (Roche, Mannheim, Germany) was used and luminescence was measured with the Infinite M200 pro plate reader attenuation setting automatic mode (Tecan Group Ltd, Männedorf, Switzerland).

### Binding affinity studies using surface acoustic wave (SAW) technology

The sam^®^5BLUE biosensor instrument (NanoTemper Technologies, Munich, Germany) was used to determine the binding affinities of the different aptamers. Rituximab (MabThera, Roche) was coupled to the surface of a sam^®^5BLUE protein A sensor chip (NanoTemper Technologies). Rituximab was diluted in 1x PBS to a concentration of 2200 nM and 200 µl of the rituximab solution were injected to the chip. Residual activated groups on the surface of the chip were blocked by injecting 125 µl of 1 M ethanolamine pH 8.5. For the calculation of the affinity constant K_d_ increasing aptamer concentrations were injected. Freshly folded aptamers (8 min at 94 °C, 15 min on ice, 10 min at RT) were applied at a concentration of 0–8000 nM in buffer composed of 20 mM Tris-HCl pH 7.6, 100 mM NaCl, 2 mM MgCl_2_, 5 mM KCl, 1 mM CaCl_2_, 0.02% v/v Tween 20 (running buffer). The coated chip was equilibrated with the identical buffer and affinity measurements were performed at 22 °C at a flow rate of 40 µl/min. Aptamers were injected for 4 min followed by a 4 min wait. Between each injection, residual aptamers were removed with regeneration buffer (running buffer including 1 M NaCl). SAW phase changes were recorded and Trace Drawer 1.7 software was used to calculate the affinities. Affinity constants and K_d_ values were determined from kinetics evaluation using the provided uncoupled 1:1 binding model.

### Secondary structure prediction and circular dichroism spectroscopy

Secondary structure prediction of aptamers was performed using Mfold (http://unafold.rna.albany.edu) at 22 °C in 100 mM Na^+^ and 2 mM Mg^2+,^^70^. The QGRS Mapper (http://bioinformatics.ramapo.edu/QGRS) was used for G-quadruplex prediction and recommended settings were employed^71^. Circular dichroism spectra were recorded using a JASCO J-815 spectropolarimeter (Jasco, Tokyo, Japan). Non-biotinylated aptamers were folded as described above. UV-spectra ranging from 200–320 nm were recorded at 20 °C and results are presented as mean residue molar ellipticity.

### Robustness test of the ELASA setup

Ten varying buffer and ten folding conditions were used to investigate the robustness of the ELASA setup (for details see Figures 3a and 4a). RA2 and RA4 were used at a concentration of 25 nM and the ELASA was performed as described above.

### Binding analysis of rituximab, Fc fragments and other biologics

Assay conditions of the ELASA were carried out as described above. The following proteins were included in the assay and captured by the coated protein A: rituximab (MabThera lot N7075B10, Roche), glycosylated Fc fragment of rituximab (MabThera lot N7075B10, Roche) were prepared using the FragIT™ Kit (Genovis, Lund, Sweden) according to the provided protocol, and Fc/2 fragment was obtained from *E. coli* as described elsewhere^34^. In addition, the IgG1 antibodies adalimumab (Humira, AbbeVie Ltd., North Chicago, US), bevacizumab (Avastin, Roche), and etanercept (Enbrel, Amgen, Thousand Oaks, US) an antibody containing the Fc part of an IgG1 antibody were tested. Aptamers RA1, RA3, RA5 and RA6 were used at a concentration of 50 nM and aptamers RA2 and RA4 at 25 nM, respectively.

### Analysis of stressed rituximab samples using an ELASA

To simulate protein stress conditions, aliquots of rituximab (MabThera, lot N7075B10) were treated as follows: ten freeze/thaw cycles, incubation at 4 °C, 25 °C and 40 °C for 72 h, exposure to UV light for 30 seconds or two minutes on an UV light box (setting 100%) and vortexing 10 times for 1 min. The respective samples were captured by protein A and analysed using the different aptamers at a concentration of 50 nM using the protocol described above.

### Comparison of MabThera, GP2013 and Reditux

Samples of the rituximab originator (MabThera, lot 1 N7075B10, lot 2 N7025B04, Roche, Basel Switzerland), the biosimilar GP2013_DP (lot 1 EH7223_ID_12_7 and lot 2 EU8142_ID_11_7, Sandoz, Basel, Switzerland) and the copy product Reditux (lot 1 RIAV2416, lot 2 RIAV2616, lot 3 RIAV0517, Dr. Reddy’s Laboratories, Hyderabad, India) were captured by the coated protein A and analysed using the different aptamers. All biologics were formulated in 25 mM sodium citrate, 0.5 mM polysorbate 80, 154 mM NaCl, pH 6.5 at a concentration of 10 mg/ml. The ELASA setup was as described above; RA1, RA3, RA5 and RA6 were used at a concentration of 50 nM and aptamers RA2 and RA4 at 25 nM, respectively. Three independent assays were performed and results were normalised for MabThera lot 1 as 100% value.

### Statistical analyses

Statistical analyses were performed in GraphPad Prism 7.03. A one-way ANOVA with corrections for multiple comparisons (Dunnett) to the control column (MabThera originator lot 1) was performed. Results with p ≤ 0.05 were considered statistically significant.

## Supplementary information

Supplementary Table and Figures

## Data Availability

All data generated or analysed during this study are included in this published article (and its Supplementary Information files).
