# Supplementary material for: Aptamers as quality control tool for production, storage and biosimilarity of the anti-CD20 biopharmaceutical rituximab
Source: Sci Rep. 2019 Feb 1;9:1111. doi: 10.1038/s41598-018-37624-1 (PMC6358617; doi:10.1038/s41598-018-37624-1)
Supplement: Supplementary file 1 — Supplementary Table and Figures [file 41598_2018_37624_MOESM1_ESM.pdf]

Aptamers as quality control tool for production, storage and biosimilarity of the anti-CD20 biopharmaceutical rituximab

Sabrina Wildner[1,2], Sara Huber[2], Christof Regl[1,2], Christian G. Huber[1,2], Urs Lohrig[3], Gabriele Gadermaier*[1,2]

[1]Christian Doppler Laboratory for Innovative Tools for Biosimilar Characterization, University of Salzburg, Hellbrunner Straße 34, 5020 Salzburg, Austria

[2]Department of Biosciences, University of Salzburg, Hellbrunner Straße 34, 5020 Salzburg, Austria

[3]Technical Development Biosimilars, Global Drug Development, Novartis, Sandoz GmbH, Biochemiestrasse 10, 6250 Kundl, Austria

***Corresponding author**

Gabriele Gadermaier

Christian Doppler Laboratory for Biosimilar Characterization

Department of Biosciences, University of Salzburg

Hellbrunnerstraße 34, A-5020 Salzburg, Austria

Telephone: 0043-662-8044-5974, Fax: 0043-662-8044-183

Gabriele.Gadermaier@sbg.ac.at

Supplementary Table S1. Overview on aptamer sequences obtained after SELEX screening.

Name	Aptamer sequence (5´-3´)	Occurrence
RA1	CGGCGGGGGGAGGATTGTGGTCTGCTCATGGCTGCCGTTT	4
RA2	TGGGGGTAGGATTGTGGTTGGCTTTAATTGCTTTGGTGGT	4
RA3	GGGGGTGAGGATTGTGGTTTGGCTTATTGGTTTGCTGGTG	12
RA4	TATACTGGGCCGTGCGTGACTTTTCCGTGCTGCATGAGAG	3
RA5	GGCCGGTAGATGGGGAATCGGTTTCGGTGGGGCTAGGGAC	2
RA6	CGTGGGTGGGGATTGTGGTTTGGCTGATGGGGTGCTGGTT	2
RA7	CCACCAGGTGGGTACGGGTCTGCAAGGTTGGATGAGGTTT	4
RA8	GTCGTGGGCGGGACTGTGGCGGTTTCTTTGGCTGCTATTG	9
RA9	TTGGGCAGGAGGGGTTGTGGCATTTGGTTGTTGGTTGTGT	4
RA10	GCCGGGCGTCGTGTATTTGTGTTTTGGTGTCCCTTGAGTG	1
RA11	GTGCCAAACTCAAGCTTTGTAAGCTATTTGCGGCTTGGTT	1
RA12	CCCCCTACCGAAGGCGCGCAGCGCACAATGTGCCGTTCAG	1
RA13	GGGGGTGAGGATTGGGGTTTGGCTTATGGGTGTGCGGGGG	1
RA14	GTGGGTACGGGCCTGCATTCCGCTATTGGGTGGTTGGTGA	1
RA15	CGGCGGGGGGAGGATCGTGGTCC-ATCATGGCTCCCGTTC	1

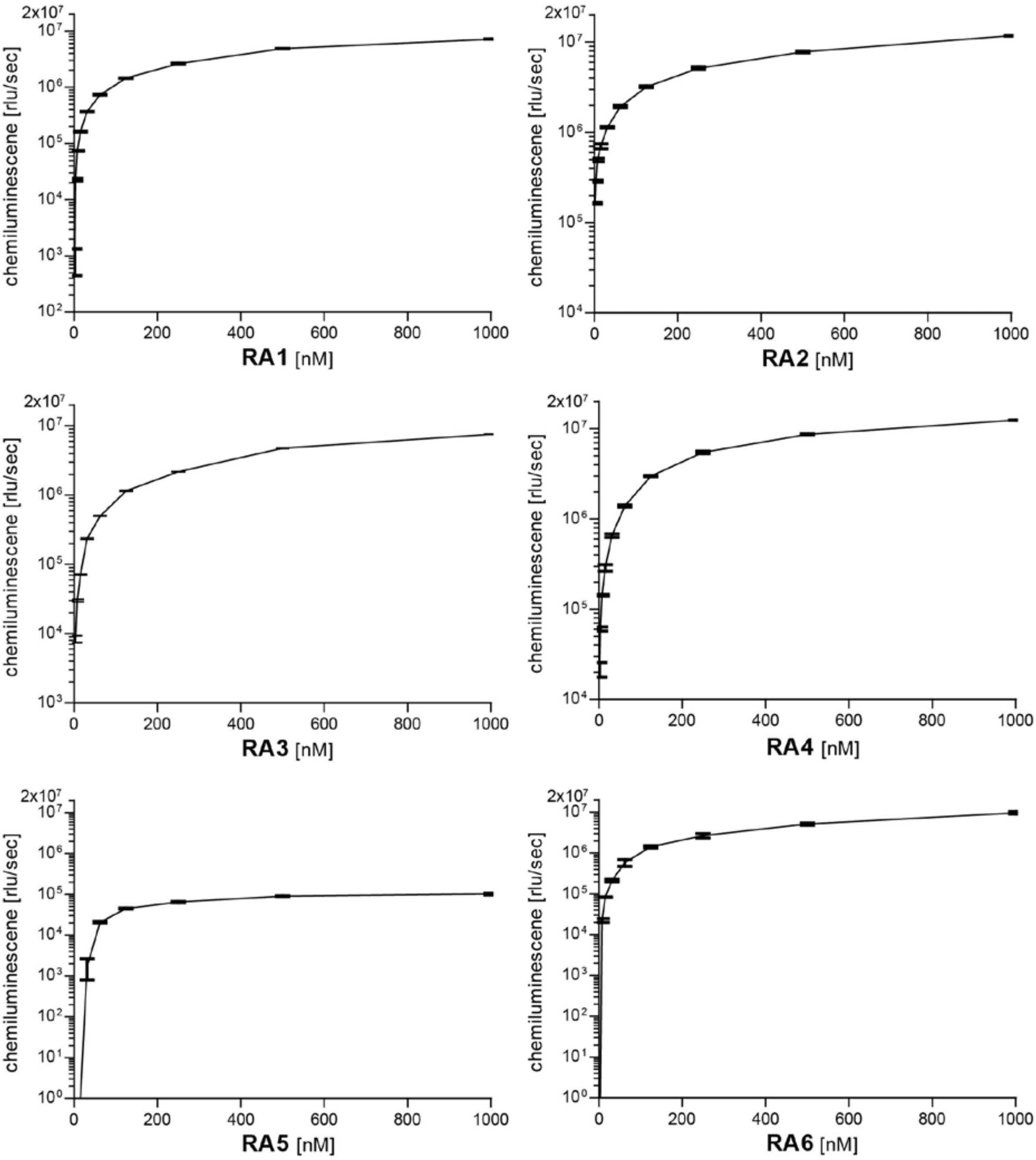

Supplementary Figure S1. Titration curves of aptamers against rituximab determined by ELASA. Serial dilutions of aptamers (1.95 nM to 1000 nM) were tested with protein A bound rituximab. Aptamers were measured in triplicates, and graphs represent mean values with standard errors.

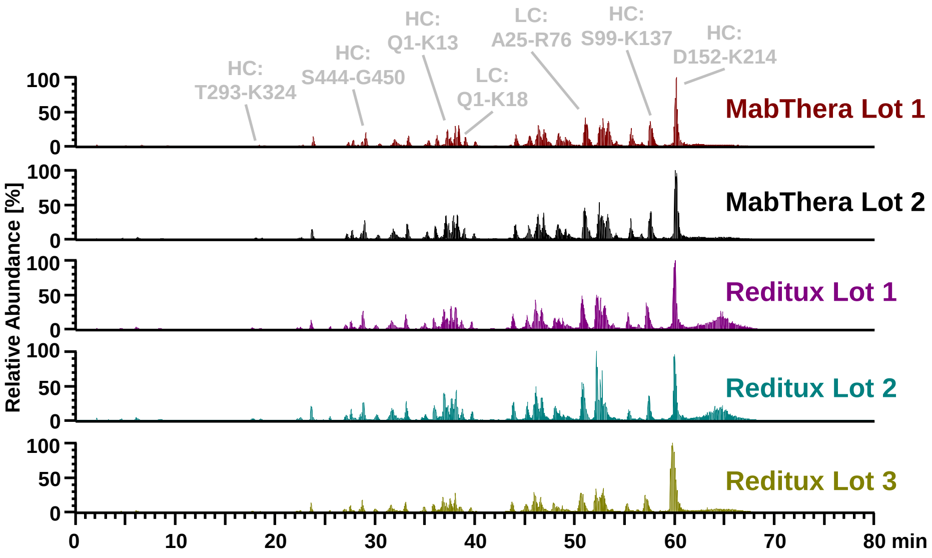

Domain	Residue #	Modification	MabThera Lot 1	MabThera Lot 2	Reditux Lot 1	Reditux Lot 2	Reditux Lot 3
Heavy chain	1	Q1+NH3 loss	**99.58 ± 0.01**	**99.89 ± 0.01**	**99.23 ± 0.1**	**98.82 ± 0.02**	**98.73 ± 0.12**
Light chain	1	Q1+NH3 loss	**93.36 ± 0.08**	**94.78 ± 0.09**	**89.08 ± 0.23**	**87.35 ± 0.34**	**89.29 ± 0.09**
Heavy chain	47	W47+Oxidation	**1.16 ± 0.07**	**1.02 ± 0.07**	**1.14 ± 0**	**1.18 ± 0.02**	**1.1 ± 0.04**
Light chain	148	K148+Glycation	**0.73 ± 0.06**	**0.7 ± 0.07**	**1.15 ± 0.05**	**1.14 ± 0.03**	**1.09 ± 0.05**
Heavy chain	256	M256+Oxidation	**1.58 ± 0.04**	**1.79 ± 0.06**	**2.81 ± 0.06**	**2.28 ± 0.02**	**1.66 ± 0.06**
Heavy chain	330	K330+Glycation	**0.56 ± 0.02**	**0.52 ± 0.03**	**0.69 ± 0.04**	**0.76 ± 0.06**	**0.68 ± 0.01**
Heavy chain	450	G450+Lys	**0.75 ± 0.03**	**0.72 ± 0.03**	**26.54 ± 0.9**	**25.82 ± 0.58**	**22.78 ± 0.71**
Heavy chain	450	G450+ProlineA midation	**0.07 ± 0**	**0.04 ± 0**	**0.03 ± 0**	**0.03 ± 0**	**0.03 ± 0**

Supplementary Figure S2. Peptide mapping and modification summary of rituximab samples after proteolytic digestion with Trypsin/Lys-C. Given are average ± standard deviation of three technical replicates. Peptides are eluting between 5.0-61.0 min, the most intense peptides and peptides with high rates of posttranslational modifications are indicated in grey. 100 µg of each rituximab were diluted to a concentration of 5.0 $mg.mL^{-1}$ in 4 $mol.L^{-1}$ GdnHCl and 5 $mmol.l^{-1}$ TCEP. Disulfides were reduced for 15 min at 60 °C and alkylated with of 20 mmol.L-1 IAA at 22 °C for 30 min. Proteolysis was performed upon dilution to a final concentration of 0.50 mg.mL-1 in 175 $mmol.L^{-1}$ ammonium acetate, addition of Trypsin-Lys-C mixture at a mAb to enzyme ratio of 25:1 (w:w) and incubation for 3.0 hours at 37 °C. Chromatographic separation was carried out on a Dionex™ UltiMate™ 3000 Rapid Separation system from Thermo Fisher Scientific (Germering, Germany) at a flow rate of 60 $µL.min^{-1}$ utilizing a Hypersil GOLD aQ C18 column (100 x 1.0 mm i.d., 1.9 µm particle size, 175 Å pore size, Thermo Fisher Scientific, Sunnyvale, CA, USA) operated at a temperature of 50 °C. Mobile phase A was composed of H2O + 0.10% FA, mobile phase B of ACN + 0.10% FA. The gradient applied was: 2.0% B for 2 min, 2.0–10.0% B in 5 min, 10.0–35.0% B in 43 min, 80% B for 5 min, and 2.0% B for 30 min. For each replicate 1 µg mAb digest was injected. Mass spectrometry was conducted on a benchtop quadrupole-Orbitrap instrument (Q Exactive™) equipped with an Ion Max™ source with a heated electrospray ionization (HESI) probe, both from Thermo Fisher Scientific (Bremen, Germany). Data acquisition was conducted using Chromeleon 7.2 (Thermo Fisher Scientific, Germering, Germany). The HESI source heater temperature was set to 100°C, spray voltage to 3.5 kV, sheath gas flow to 10 arbitrary units, auxiliary gas flow of 5 arbitrary units, capillary temperature to 300 °C and S-lens RF level to 60.0. Each scan cycle consisted of a full scan at a scan range of m/z 300–2,000 and a resolution setting of 70,000, followed by 10 data-dependent HCD scans at 28 NCE at a resolution setting of 17,500. Dynamic exclusion was set to 15 seconds. Deconvolution and assignment of mass spectra was done with BioPharma Finder 3.0 (Thermo Fisher Scientific, Sunnyvale, CA, USA) with the following settings: 10 ppm mass accuracy, minimum confidence of 0.8, specificity 'high' for trypsin protease and the following variable modifications: built-in N-glycan library for chinese hamster ovary cell lines, N-terminal pyro-glutamate (Q), C-terminal lysine variants and proline-amidation, oxidation (M, W), deamidation (N, Q), glycation (K), carbamidomethylation, NH3-loss, and H2O-loss, maximum allowed number of variable modifications of 3 per peptide.
